# The Complete Mitochondrial Genome of the Freshwater Fish *Onychostoma ovale* (Cypriniformes, Cyprinidae): Genome Characterization and Phylogenetic Analysis

**DOI:** 10.3390/genes14061227

**Published:** 2023-06-06

**Authors:** Renyi Zhang, Tingting Zhu, Qi Luo

**Affiliations:** School of Life Sciences, Guizhou Normal University, Guiyang 550025, China; ztingting0115@163.com (T.Z.); luoqi685@163.com (Q.L.)

**Keywords:** Acrossocheilinae, *Onychostoma ovale*, mitogenome, phylogeny

## Abstract

In this study, we sequenced and characterized the complete mitochondrial genome (mitogenome) of *Onychostoma ovale*. The mitogenome of *O. ovale* was 16,602 bp in length with 13 protein-coding genes (PCGs), 22 transfer RNA (tRNA) genes, 2 ribosomal RNA (rRNA) genes, and a control region. The nucleotide composition of the *O. ovale* mitogenome was 31.47% A, 24.07% T, 15.92% G, and 28.54% C, with a higher A + T content (55.54%) than G + C content (44.46%). All PCGs began with the standard ATG codon, except for the cytochrome c oxidase subunit 1 (*COX1*) gene and the NADH dehydrogenase 3 (*ND3*) gene with GTG, while six PCGs ended with incomplete termination codons (TA or T). The Ka/Ks ratios of 13 PCGs were all less than one, indicating that they were under purifying selection. All tRNA genes were folded into the typical cloverleaf secondary structures with the exception of *tRNA^Ser(AGY)^*, whose dihydrouridine (DHU) arm was absent. The phylogenetic trees showed that *Onychostoma* and *Acrossocheilus* were classified into three clades. There was a mosaic relationship between *Onychostoma* and *Acrossocheilus*. Moreover, the phylogenetic tree analysis showed that *O. rarum* was the closest species to *O. ovale*. This study can provide a useful resource for further phylogeny and population genetic analyses of *Onychostoma* and *Acrossocheilus*.

## 1. Introduction

In fish, as in other vertebrates, mitochondrial DNA (mtDNA) is organized as an extranuclear, closed circular, double-stranded DNA molecule that is composed of the heavy (H) strand and the light (L) strand) [1,2]. Fish mtDNA is generally small, ranging from 15–18 kb, which typically contain 13 protein-coding genes (PCGs), 22 transfer RNA (tRNA) genes, 2 ribosomal RNA (rRNA) genes, and 1 control region (D-loop) [2,3]. In comparison with nuclear DNA, mtDNA has the unique characteristics of maternal inheritance, multiple copies, no introns, a rapid evolution rate, and small molecular size, so it has become an important molecular marker in evolutionary genetics, molecular ecology, species identification, and the conservation biology of fish [1,4,5]. In recent years, with the rapid development and application of high-throughput DNA sequencing technologies and bioinformatics analysis, more and more fish mitochondrial genomes have been successfully sequenced and characterized.

The newly established subfamily Acrossocheilinae consists of three genera (*Acrossocheilus*, *Onychostoma*, and *Folifer*) distributed in East Asia and Southeast Asia, including Vietnam, Laos, Thailand, Cambodia, and China [6,7]. The subfamily consists of 23 species in the genus *Onychostoma*, 26 species in the genus *Acrossocheilus*, and only 3 valid species in the genus *Folifer* [8]. It is a kind of small- and medium-sized freshwater economic fish [9]. Acrossocheilinae is characterized primarily by three rows of pharyngeal teeth, a dorsal fin with eight branched rays, a lower jaw with a horny sheath, and the last simple ray of the dorsal fin with a serrated or smooth posterior edge [6]. Previous molecular phylogenetic studies have shown that Acrossocheilinae is a monophyletic group [6,10]. However, the phylogenetic relationship between *Acrossocheilus* and *Onychostoma* has long been controversial [6,11]. More extensive species sampling will be essential to refine our understanding of the molecular phylogeny of Acrossocheilinae. So far, there are a total of 28 available mitogenomes of the subfamily Acrossocheilinae at the National Center for Biotechnology Information.

*Onychostoma ovale* Pellegrin & Chevey, 1936 is a kind of medium-sized freshwater fish species that is distributed in the Yuanjiang River in China and can also be found in the Pearl River and Wujiang River. *O*. *ovale* is a bottom-dwelling freshwater fish and feeds mainly on algae and copepods [12]. *O*. *ovale* can be distinguished from other *Onychostoma* fish based on its morphological characteristics such as the number of lateral line scales and the length of the first branch of the dorsal fin [9]. In this study, we sequenced and analyzed the mitogenome of *O*. *ovale* and reconstructed the mitogenomic phylogeny of *Onychostoma* and the relative genera (*Acrossocheilus* and *Folifer*) with 13 PCGs. The results of this study may provide basic genetic information for phylogenetic and population genetic studies of the Acrossocheilinae and expand our knowledge of the mitochondrial genome features of Acrossocheilinae and the classification of the subfamily Acrossocheilinae.

## 2. Materials and Methods

### 2.1. Sampling, DNA Extraction, PCR Amplification, and Sanger Sequencing

An individual sample of *O. ovale* was collected in Luodian Country, Guizhou Province, China (25°33′ N, 106°51′ E). The voucher specimen (GZNUSLS202009033) was preserved immediately in 75% ethanol and then stored at −20 °C for genomic DNA extraction. Total genomic DNA was extracted from a piece of muscle tissue using a modification of the high salt method [13] and standard protease K digestion. The integrity of the extracted DNA was checked by 1% agarose gel electrophoresis, and the DNA concentration and purity were determined by analysis with the Epoch 2 spectrophotometer system (Biotek Instruments, Inc., Winooski, VT, USA). The genomic DNA was used as a template for the overlapping polymerase chain reaction (PCR) amplification. Thirteen pairs of PCR primers were designed according to the conservative region of *O. rarum* (GenBank accession number: NC_022869) (Table 1). The PCR was performed in a total volume of 35 μL containing 17.5 μL of 2xTaq Plus MasterMix (CoWin Biosciences, Beijing, China), 14.5 μL ultrapure water, 1 μL of template DNA (100 ng/μL), and 1 μL of each primer (10 μM). The PCR conditions were as follows: initial denaturation for 5 min at 95 °C followed by 35 cycles of 1 min denaturation at 95 °C, 30 s annealing at 37–53.4 °C (Table 1), and 1.5 min elongation at 72 °C, with a final extension for 10 min at 72 °C. The amplified PCR products were visualized on 1% agarose gels to confirm amplification. The sizes of the amplified PCR products were estimated by comparison to a DL2000 DNA size marker (TaKaRa, Beijing, China). The PCR products were then sent to Sangon Biotech Company (Shanghai, China) and sequenced directly on both strands using the same primers for PCR amplification with a 3730xl DNA analyzer (Applied Biosystems, Thermo Fisher Scientific, Waltham, MA, USA) and a BigDye Terminator v3.1 Cycle Sequencing Kit (Applied Biosystems).

### 2.2. Mitogenome Assembly, Annotation, and Bioinformatics Analysis

The sequence fragments were manually assembled into a circularized contig by SeqMan software (DNA STAR package; DNAStar Inc., Madison, WI, USA). After being assembled, the mitogenome sequence was automatically annotated using the MitoAnnotator pipeline [14]. In addition, the tRNA genes were identified and annotated using MITOS Web Server [15] and tRNAscan-SE search server [16]. The sequences of the extend termination associated sequence (ETAS), the central conserved blocks (CSB-F, -E, -D), and the conserved sequence block domains (CSB-1, -2, -3) in the control region were identified using the Basic Local Alignment Search Tool (BLAST) against the sequences of the reported fish. The base composition and codon usages were calculated using MEGA 6.0 software [17]. The relative synonymous codon usage (RSCU) of each PCGs was analyzed using PhyloSuite v1.2.3 [18]. We calculated A + T skew and G + C skew using the following general formulae: A + T skew = (A% − T%)/(A% + T%) and G + C skew = (G% − C%)/(G% + C%), respectively [19]. The rates of non-synonymous substitutions and synonymous substitutions (Ka/Ks) in the mitogenomes of all species of Acrossocheilinae were calculated using DnaSP 6.0 [20].

### 2.3. Phylogenetic Analysis

We herein reconstruct the phylogeny of the Acrossocheilinae using the mitogenome sequences of 29 species (Table 2); *Cyprinus carpio* was used as an outgroup (Table 2). The nucleotide sequences of 13 PCGs of all mitogenomes were extracted by PhyloSuite v1.2.3 [18]. Then, sequences were aligned using MAFFT v7.313 [21] with default parameters in PhyloSuite v1.2.3 [18] and concatenated into a single supergene for each species. The optimal partitioning strategy and the best-fit evolution model for each partition were inferred using PartitionFinder v2 [22] under the Bayesian information criterion (BIC) and the greedy search scheme in PhyloSuite v1.2.3 [18]. The phylogenetic relationships of 29 species of the subfamily Acrossocheilinae were inferred using the maximum likelihood (ML) and Bayesian inference (BI) methods based on the concatenated nucleotide sequences of all 13 PCGs. The ML phylogenetic tree was constructed using IQ-TREE v. 1.6.8 [23] in PhyloSuite v1.2.3 [18] with 1000 bootstrap replicates. The BI analysis was performed with MrBayes v. 3.2.6 [24]. Four Markov chain Monte Carlo (MCMC) chains were run simultaneously for 20 million generations sampling every 1000 generations. Bayesian posterior probability (BPP) was calculated in a majority-rule consensus tree after discarding the first 25% of samples as burn-in. The phylogenetic trees were visualized and edited using the online tool Interactive Tree Of Life (iTOL) (https://itol.embl.de/) (accessed on 19 January 2023) [25].

## 3. Results and Discussion

### 3.1. Mitochondrial Genomic Structure and Base Composition

**Figure 1:**
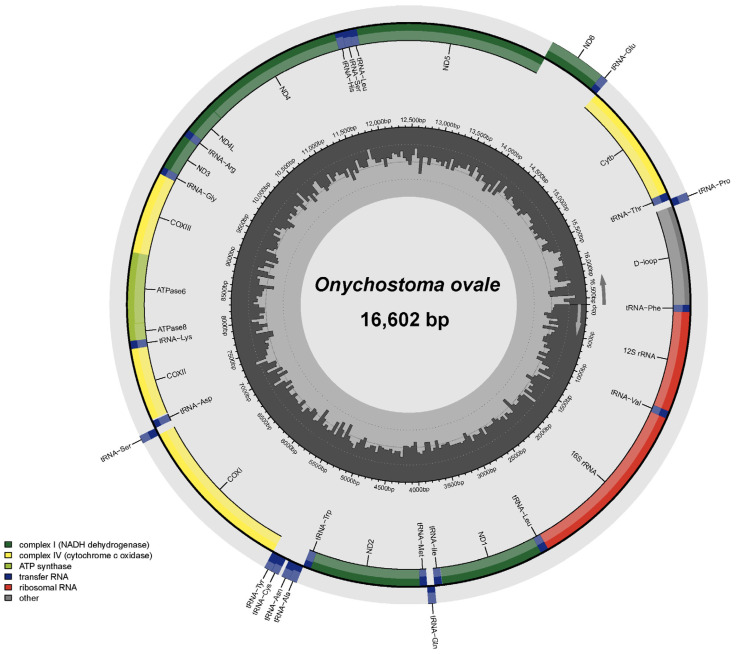
Circular map of the mitogenome of *O. ovale*.

The complete mitogenome sequence of *O. ovale* (GenBank accession number: NC_066040) was 16,602 bp (Figure 1), which was consistent with other known species of Acrossocheilinae (Table 2). Like most fish, the mitogenome of *O. ovale* also contained 37 mitochondrial genes, with 13 typical PCGs, 22 tRNA genes, 2 rRNA genes, and 1 control region (Figure 1; Table 3). All mitochondrial genes were encoded on the H chain, with the exception of the *ND6* gene and the eight tRNA genes (*tRNA^Gln^*, *tRNA^Ala^*, *tRNA^Asn^*, *tRNA^Cys^*, *tRNA^Tyr^*, *tRNA^Ser^*, *tRNA^Glu^*, and *tRNA^Pro^*) were encoded on the L chain (Figure 1; Table 3). In the mitogenome, there were six overlapping regions (from 1 to 7 bp in size). The longest overlapping regions (7 bp) were located between *ATP8*/*ATP6* and *ND4L*/*ND4*. In addition, there were 12 gene spacers (from 1 to 33 bp in size). The longest gap was found between *tRNA^Asn^* and *tRNA^Cys^* by 33 nucleotides.

The overall base composition of *O. ovale* mitogenome was slightly biased toward A and T at 55.54% (A = 31.47%%, T = 24.07%, G = 15.92%, and C = 28.54%) with a positive A + T skew (0.13) and a negative G + C skew (−0.28) (Table 4). The mitogenome of *O. ovale* exhibited a clear A + T preference in its base composition, which was similar to that of other *Onychostoma* fish (Table 2). Compared with the whole genome, the control region has the highest A + T content, up to 66.99% (Table 4), which is a typical feature of animal mitochondrial genomes [2,26]. On the contrary, the first codon position of the PCGs with the lowest A + T content, which was 47.10% (Table 4). 

### 3.2. Protein-Coding Genes and Codon Usage

The PCGs ranged in size from 165 bp (*ATP8*) to 1824 bp (*ND5*) with a total length of 11,410 bp. Eleven PCGs were canonical ATG start codons, while the start codon of the *COI* gene, and the *ND3* gene was a GTG start codon. The non-standard start codon was also found in other fish species [4,5]. Seven PCGs had complete stop codons, while the remaining six PCGs ended with the incomplete stop codons TA or T (*COIII* ended with TA and *ND2*, *COII*, *ND3*, *ND4*, and *Cyt b* ended with T) (Table 3). These incomplete stop codons widely exist in vertebrate mitochondrial PCGs, which were presumed to be completed via post-transcriptional polyadenylation [27]. Moreover, the values of A + T skew and G + C skew for the PCGs were 0.07 and –0.31, respectively, indicating that the abundance of A and C is higher than that of their respective counterparts (Table 4).

**Figure 2:**
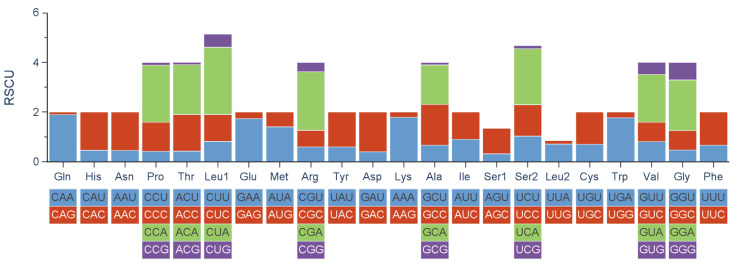
Relative synonymous codon usage of all PCGs in the mitogenome of *O. ovale*.

The amino acid usage and RSCU values in the PCGs of *O. ovale* are summarized in Table 5 and Figure 2. The mitogenome encoded a total of 3801 amino acids, among which leucine (16.55%) and cysteine (0.68%) were the most and the least frequently used amino acids, respectively. The six most frequently used codons in *O. ovale* were CUA (Leu), ACA (Thr), AUC (Ile), UUC (Phe), GCC (Ala), and GCA (Ala).

### 3.3. Selective Pressure Analysis

**Figure 3:**
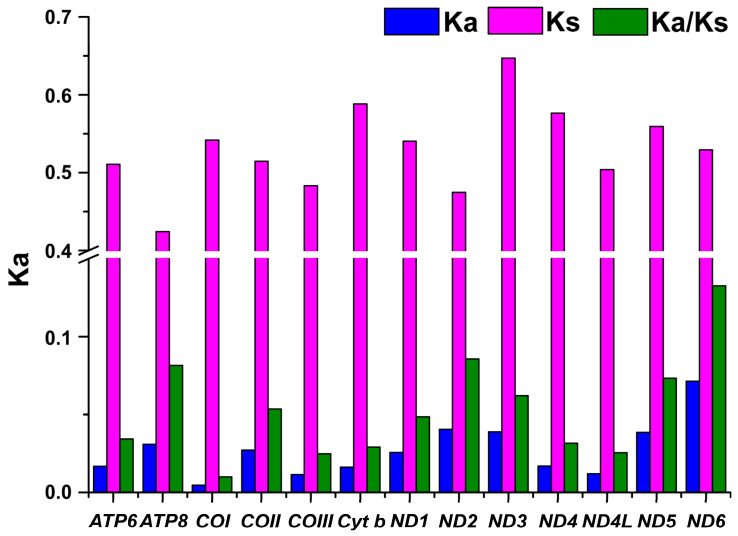
The Ka, Ks, and Ka/Ks values for each PCG from 29 Acrossocheilinae species mitogenomes.

In order to investigate the selective pressure on 13 PGCs of 29 Acrossocheilinae species, we calculated the non-synonymous substitutions rate (Ka) to the synonymous substitutions rate (Ks) ratio (Ka/Ks). The Ka/Ks ratios of all PCGs were far lower than one (Figure 3), indicating that all of the PCGs were evolving under strong purifying selection in these species [28]. The *ND6* gene exhibited the highest ratio (Ka/Ks = 0.133) of all the PCGs, whereas the *COI* gene had the lowest ratio (Ka/Ks = 0.01).

### 3.4. Transfer RNAs, Ribosomal RNAs, and Control Region

The mitogenome of *O. ovale* consisted of 22 tRNA genes individually ranging in size from 67 to 76 bp, representing 9.4% (1563 bp) of the entire mitogenome (Table 3). Among the 22 tRNA genes, 14 tRNA genes were encoded on the H strand, while 8 tRNA genes were encoded on the L strand (Table 3), and this distribution was similar to that observed in other Acrossocheilus species [29,30]. All tRNA genes were predicted to fold into the typical cloverleaf secondary structures except that the *tRNA^Ser(AGY)^* lacked the dihydrouridine (DHU) arm (Figure S1), which has been reported in most bony fish [31,32]. The A + T content of the 22 tRNA genes was 55.60%, with a positive A + T skew (0.03) and G + C skew (0.05).

There were two rRNA genes, a small ribosomal RNA (12S rRNA) gene and a large ribosomal RNA (16S rRNA) gene. The lengths of the 12S rRNA gene and the 16S rRNA gene were 959 bp and 1680 bp, respectively (Table 3). As in other vertebrates, they were located between *tRNA^Phe^* and *tRNA^Leu^* and were separated from each other by *tRNA^Val^* (Figure 1). The A + T and G + C content of the two rRNA genes was 53.69% and 46.31% and the A + T skew and G + C skew were 0.29 and −0.10, respectively, suggesting an apparent bias toward the use of A and C.

The control region in the mitogenome is also known as the A + T-rich region and is essential for the initiation of mitogenome replication and transcription [26,33]. The control region of *O. ovale* is located between *tRNA^Phe^* and *tRNA^Pro^*, with a total length of 945 bp (Figure 1; Table 3). An extend terminal associated sequence (ETAS), central conserved sequence block (CSB) domains containing three conserved sequence blocks (CSB-F, CSB-E, and CSB-D), and a variable domain consisting of three conserved sequence blocks (CSB-1, CSB-2, and CSB-3) were identified in the control region of *O. ovale* through a homology search (Figure S2).

### 3.5. Phylogenetic Analysis

**Figure 4:**
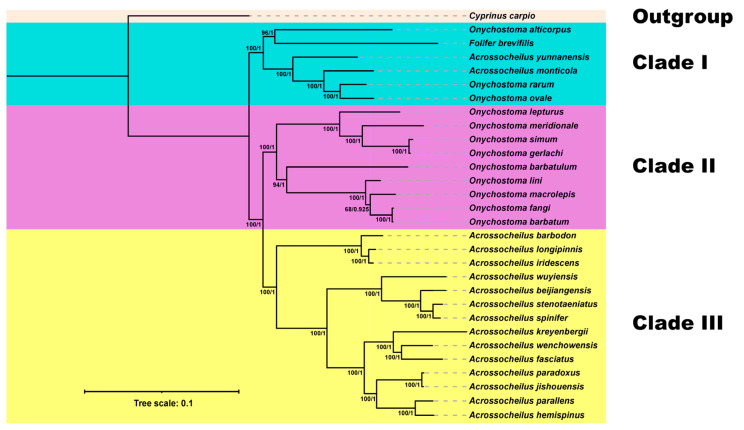
Phylogenetic estimate of relationships within the subfamily Acrossocheilinae based on the 13 PCGs using Bayesian inference (BI) and maximum likelihood (ML) analyses. The BI posterior probability (right) and ML bootstrap support values (left) are denoted at each node.

The molecular phylogenetic trees of Acrossocheilinae were reconstructed using both ML and BI methods on the 13 concatenated protein-coding genes. Phylogenetic analyses inferred through BI and ML yielded a consistent topology. The phylogenetic trees showed that Acrossocheilinae could be divided into three distinct clades (Figure 4), which is congruent with previous studies [6,11]. Clade I included both *Onychostoma* (*O. alticorpus*, *O. rarum*, and *O. ovale*), *Acrossocheilus* (*A. monticola* and *A. yunnanensis*), and *Folifer* (*F. brevifilis*) (Figure 4). Clade II was composed of nine *Onychostoma* species. Clade III was composed of 14 *Acrossocheilus* species. The phylogenetic trees showed that *O. ovale* was most closely related to *O. rarum*, and they were grouped with other species belonging to clade I with high bootstrap support. Our phylogenetic results also supported that neither *Onychostoma* nor *Acrossocheilus* was a monophyletic group [11]. The phylogenetic trees suggested that the classification of *Onychostoma* and *Acrossocheilus* should be further evaluated and revised. The current molecular phylogenetic study of *Onychostoma* showed that it was a complex group. This implied a conflict between morphological and molecular phylogenetic classification. Therefore, in future studies, extensive taxon sampling and new types of molecular markers are needed. 

## 4. Conclusions

We obtained the mitogenome sequence of *O. ovale* by overlapping PCR, and its length was 16,602 bp. The mitogenome was composed of 37 genes (13 PCGs, 22 tRNA genes, and 2 rRNA genes) and a control region. The genome size, gene arrangement, codon usage, and nucleotide composition of *O. ovale* were similar to those of other fish reported previously. The mitogenome of *O. ovale* showed a clear A + T preference in base composition. Most of the PCGs started with the standard ATG codon and stopped with the termination codon TAA. Moreover, the Ka/Ks ratios were all less than 1, indicating that the PCGs of these Acrossocheilinae species were under purifying selection. The phylogenetic trees showed that *Onychostoma* and *Acrossocheilus* were classified into three clades. The results of this study can provide valuable genetic data for population genetic studies and phylogenetic analysis of *Onychostoma* and *Acrossocheilus*.

## Figures and Tables

**Table 1:** Thirteen PCR primers for the amplification of the mitochondrial genome of *O. ovale*.

Primer Name	Primer Sequences (5′–3′)	Annealing Temperature
OF1	AGGGACAAAAGTAAGCAAAA	43.7 °C
OR1	CCCAACCGAAGGTAAAATA	
OF2	TGCCCAGTGACCACAAGTT	51.6 °C
OR2	GTGAGGCTCCCAGGAAAAG	
OF3	GTGAGGCTCCCAGGAAAAG	43.9 °C
OR3	TGGTTGAGTTGGTTGTGTT	
OF4	TTAGTAGGGGGATGAGGAG	47.3 °C
OR4	GGGTCAAAGAATGTGGTGT	
OF5	TTCCACGAATGAACAACA	42.2 °C
OR5	AATACAGCGGGTAAAATG	
OF6	GCATTCGTTCAAGTTCAA	44.2 °C
OR6	TACGGCAGTAGCGATAAG	
OF7	AGAAGGACACAAATGAGCAC	46.3 °C
OR7	AGGAAAAAGCGTAGAGAGAA	
OF8	GCCTGATACTGACACTTCGT	48 °C
OR8	GGCTTCTACATGTGCTTTTG	
OF9	TTCCAACCCTCATCATCAT	46.9 °C
OR9	CCTACTCCTTCTCAGCCAA	
OF10	CTTTCTCATCCTACTCCACC	45 °C
OR10	GTTTTTGCCATAGTTTTTTG	
OF11	AAGCAAACAAGTAAAAATCA	37 °C
OR11	AACAAACGGTAGTAGGAAGT	
OF12	CCTCTACAAAGAAACCTGAAAC	43.3 °C
OR12	CAAGTGAAAAGAAACCAAAAA	
OF13	TCAGGGACAATAACTGTGGGGG	53.4 °C
OR13	TTGGTGTGTTTTGACGGGGAG	

**Table 2:** Species information used in this study.

NO.	Species	Size (bp)	A%	T%	G%	C%	A + T Content	A + T Skew	G + C Skew	Accession No.
1	*Acrossocheilus barbodon*	16,596	31.55	24.37	15.88	28.20	55.92	0.13	−0.28	NC_022184
2	*Acrossocheilus beijiangensis*	16,600	31.16	24.99	16.13	27.73	56.14	0.11	−0.26	NC_028206
3	*Acrossocheilus fasciatus*	16,589	30.85	24.87	16.54	27.75	55.71	0.11	−0.25	NC_023378
4	*Acrossocheilus hemispinus*	16,590	31.16	24.70	16.15	28.00	55.85	0.12	−0.27	NC_022183
5	*Acrossocheilus iridescens*	16,596	31.51	24.42	15.93	28.14	55.94	0.13	−0.28	NC_031551
6	*Acrossocheilus jishouensis*	16,587	31.16	25.09	16.19	27.55	56.25	0.11	−0.26	NC_034917
7	*Acrossocheilus kreyenbergii*	16,849	31.16	25.43	16.26	27.15	56.60	0.10	−0.25	NC_024844
8	*Acrossocheilus longipinnis*	16,593	31.54	24.39	15.95	28.12	55.93	0.13	−0.28	NC_047455
9	*Acrossocheilus monticola*	16,599	31.42	24.54	15.81	28.24	55.96	0.12	−0.28	NC_022145
10	*Acrossocheilus paradoxus*	16,586	31.13	25.10	16.24	27.52	56.23	0.11	−0.26	MG878098
11	*Acrossocheilus parallens*	16,592	30.97	24.70	16.33	28.00	55.67	0.11	−0.26	NC_026973
12	*Acrossocheilus spinifer*	16,591	31.24	24.71	16.07	27.97	55.95	0.12	−0.27	NC_034918
13	*Acrossocheilus stenotaeniatus*	16,594	31.22	24.70	16.07	28.02	55.91	0.12	−0.27	NC_024934
14	*Acrossocheilus wenchowensis*	16,591	31.05	24.97	16.32	27.67	56.02	0.11	−0.26	NC_020145
15	*Acrossocheilus wuyiensis*	16,594	31.12	24.71	16.16	28.01	55.83	0.11	−0.27	NC_034919
16	*Acrossocheilus yunnanensis*	16,588	31.39	24.66	16.12	27.83	56.05	0.12	−0.27	NC_028527
17	*Cyprinus carpio*	16,575	31.86	24.88	15.80	27.46	56.74	0.12	−0.27	NC_001606
18	*Folifer brevifilis*	16,707	31.08	24.63	16.36	27.93	55.71	0.12	−0.26	NC_031606
19	*Onychostoma alticorpus*	16,607	30.88	23.57	16.56	28.99	54.45	0.13	−0.27	NC_021473
20	*Onychostoma barbatulum*	16,597	31.45	25.15	16.00	27.40	56.60	0.11	−0.26	NC_021644
21	*Onychostoma barbatum*	16,592	31.54	24.49	15.94	28.04	56.03	0.13	−0.28	NC_019630
22	*Onychostoma fangi*	16,597	31.55	24.50	15.90	28.05	56.05	0.13	−0.28	NC_031529
23	*Onychostoma gerlachi*	16,601	31.38	24.24	16.09	28.29	55.62	0.13	−0.27	NC_026549
24	*Onychostoma lepturus*	16,601	31.31	23.89	16.16	28.64	55.20	0.13	−0.28	NC_054158
25	*Onychostoma lini*	16,595	31.62	24.57	15.87	27.94	56.19	0.13	−0.28	NC_018043
26	*Onychostoma macrolepis*	16,595	31.29	24.53	16.21	27.97	55.82	0.12	−0.27	NC_023799
27	*Onychostoma meridionale*	16,595	31.19	24.30	16.23	28.29	55.49	0.12	−0.27	NC_031603
28	*Onychostoma ovale*	16,602	31.47	24.07	15.92	28.54	55.54	0.13	−0.28	NC_066040
29	*Onychostoma rarum*	16,590	31.49	24.15	15.88	28.47	55.65	0.13	−0.28	NC_022869
30	*Onychostoma simum*	16,601	31.31	24.30	16.11	28.28	55.61	0.13	−0.27	NC_021972

**Table 3:** Mitochondrial genome composition and characteristics of *O. ovale*.

Gene	Position Number		Size (bp)	Codon		Strand	Intergenetic Nucleotide
	Start	Stop		Start	Stop		
*tRNA^Phe^*	1	69	69			H	0
*12S rRNA*	70	1028	959			H	0
*tRNA^Val^*	1029	1100	72			H	0
*16S rRNA*	1101	2780	1680			H	0
*tRNA^Leu^*	2781	2856	76			H	0
*ND1*	2858	3832	975	ATG	TAA	H	+1
*tRNA^Ile^*	3837	3908	72			H	+4
*tRNA^Gln^*	3907	3977	71			L	−2
*tRNA^Met^*	3979	4047	69			H	+1
*ND2*	4048	5092	1045	ATG	T--	H	0
*tRNA^Trp^*	5093	5163	71			H	0
*tRNA^Ala^*	5166	5234	69			L	+2
*tRNA^Asn^*	5236	5308	73			L	+1
*tRNA^Cys^*	5342	5408	67			L	+33
*tRNA^Tyr^*	5408	5478	71			L	−1
*COI*	5480	7030	1551	GTG	TAA	H	+1
*tRNA^Ser^*	7031	7101	71			L	0
*tRNA^Asp^*	7104	7175	72			H	+2
*COII*	7191	7881	691	ATG	T--	H	+15
*tRNA^Lys^*	7882	7957	76			H	0
*ATPase8*	7959	8123	165	ATG	TAG	H	+1
*ATPase6*	8117	8800	684	ATG	TAA	H	−7
*COIII*	8800	9584	785	ATG	TA-	H	−1
*tRNA^Gly^*	9585	9656	72			H	0
*ND3*	9657	10,005	349	GTG	T--	H	0
*tRNA^Arg^*	10,006	10,075	70			H	0
*ND4L*	10,076	10,372	297	ATG	TAA	H	0
*ND4*	10,366	11,746	1381	ATG	T--	H	−7
*tRNA^His^*	11,747	11,815	69			H	0
*tRNA^Ser^*	11,816	11,884	69			H	0
*tRNA^Leu^*	11,886	11,958	73			H	+1
*ND5*	11,959	13,782	1824	ATG	TAA	H	0
*ND6*	13,779	14,300	522	ATG	TAA	L	−4
*tRNA^Glu^*	14,301	14,369	69			L	0
*Cyt b*	14,376	15,516	1141	ATG	T--	H	+6
*tRNA^Thr^*	15,517	15,588	72			H	0
*tRNA^Pro^*	15,588	15,657	70			L	−1
*D-loop*	15,658	16,602	945				0

**Table 4:** Base composition of the *O. ovale* mitochondrial genome.

	Size (bp)	A%	T%	G%	C%	A + T%	G + C%	A + T Skew	G + C Skew
Genome	16,602	31.47	24.07	15.92	28.54	55.54	44.46	0.13	−0.28
PCGs	11,410	29.36	25.74	15.45	29.45	55.10	44.90	0.07	−0.31
First codon position	3807	26.87	20.23	25.64	27.26	47.10	52.90	0.14	−0.03
Second codon position	3802	18.52	40.19	13.62	27.67	58.71	41.29	−0.37	−0.34
Third codon position	3801	42.70	16.81	7.08	33.41	59.51	40.49	0.43	−0.65
rRNA	2639	34.71	18.98	20.73	25.58	53.69	46.31	0.29	−0.10
tRNA	1563	28.60	27.00	23.22	21.18	55.60	44.38	0.03	0.05
D-loop region	945	33.97	33.02	12.59	20.42	66.99	33.01	0.01	−0.24

**Table 5:** Codon number and RSCU of *O. ovale* mitochondrial PCGs.

AA	Codon	Count	RSCU	AA	Codon	Count	RSCU
Phe	UUU(F)	74	0.66	Tyr	UAU(Y)	34	0.58
Phe	UUC(F)	149	1.34	Tyr	UAC(Y)	83	1.42
Leu	UUA(L)	73	0.70	Stop codon	UAA	6	3.43
Leu	UUG(L)	16	0.15	Stop codon	UAG	1	0.57
Leu	CUU(L)	85	0.81	His	CAU(H)	23	0.45
Leu	CUC(L)	114	1.09	His	CAC(H)	80	1.55
Leu	CUA(L)	284	2.71	Gln	CAA(Q)	99	1.89
Leu	CUG(L)	57	0.54	Gln	CAG(Q)	6	0.11
Ile	AUU(I)	128	0.89	Asn	AAU(N)	27	0.44
Ile	AUC(I)	159	1.11	Asn	AAC(N)	95	1.56
Met	AUA(M)	118	1.40	Lys	AAA(K)	69	1.79
Met	AUG(M)	50	0.60	Lys	AAG(K)	8	0.21
Val	GUU(V)	44	0.80	Asp	GAU(D)	14	0.39
Val	GUC(V)	44	0.80	Asp	GAC(D)	58	1.61
Val	GUA(V)	106	1.92	Glu	GAA(E)	87	1.74
Val	GUG(V)	27	0.49	Glu	GAG(E)	13	0.26
Ser	UCU(S)	40	1.03	Cys	UGU(C)	9	0.69
Ser	UCC(S)	49	1.26	Cys	UGC(C)	17	1.31
Ser	UCA(S)	88	2.26	Trp	UGA(W)	106	1.77
Ser	UCG(S)	5	0.13	Trp	UGG(W)	14	0.23
Pro	CCU(P)	22	0.41	Arg	CGU(R)	11	0.58
Pro	CCC(P)	63	1.18	Arg	CGC(R)	13	0.68
Pro	CCA(P)	123	2.30	Arg	CGA(R)	45	2.37
Pro	CCG(P)	6	0.11	Arg	CGG(R)	7	0.37
Thr	ACU(T)	33	0.42	Ser	AGU(S)	12	0.31
Thr	ACC(T)	117	1.48	Ser	AGC(S)	40	1.03
Thr	ACA(T)	159	2.01	Stop codon	AGA	0	0.00
Thr	ACG(T)	8	0.10	Stop codon	AGG	0	0.00
Ala	GCU(A)	55	0.65	Gly	GGU(G)	28	0.46
Ala	GCC(A)	141	1.66	Gly	GGC(G)	48	0.79
Ala	GCA(A)	135	1.59	Gly	GGA(G)	125	2.05
Ala	GCG(A)	8	0.09	Gly	GGG(G)	43	0.70

## Data Availability

The mitogenome was deposited at NCBI with the accession number NC_066040.

## Supplementary Materials

The following supporting information can be downloaded at: https://www.mdpi.com/article/10.3390/genes14061227/s1. Figure S1: Secondary structure of the 22 tRNA genes of the mitochondrial genome of *O. ovale*; Figure S2: The structure and sequence of the control region of the *O. ovale* mitochondrial genome. The extend termination associated sequence domain (ETAS), the central conserved blocks (CSB-F, CSB-E, and CSB-D), and the conserved sequence block domains (CSB-1, CSB-2, and CSB-3) were identified and are shown in red font size, and the conserved sequences are shown underlined and with black font.
