# Supplementary figures and images for: The Complete Mitochondrial Genome of the Freshwater Fish Onychostoma ovale (Cypriniformes, Cyprinidae): Genome Characterization and Phylogenetic Analysis
Source: Genes (Basel). 2023 Jun 6;14(6):1227. doi: 10.3390/genes14061227 (PMC10298739; doi:10.3390/genes14061227)

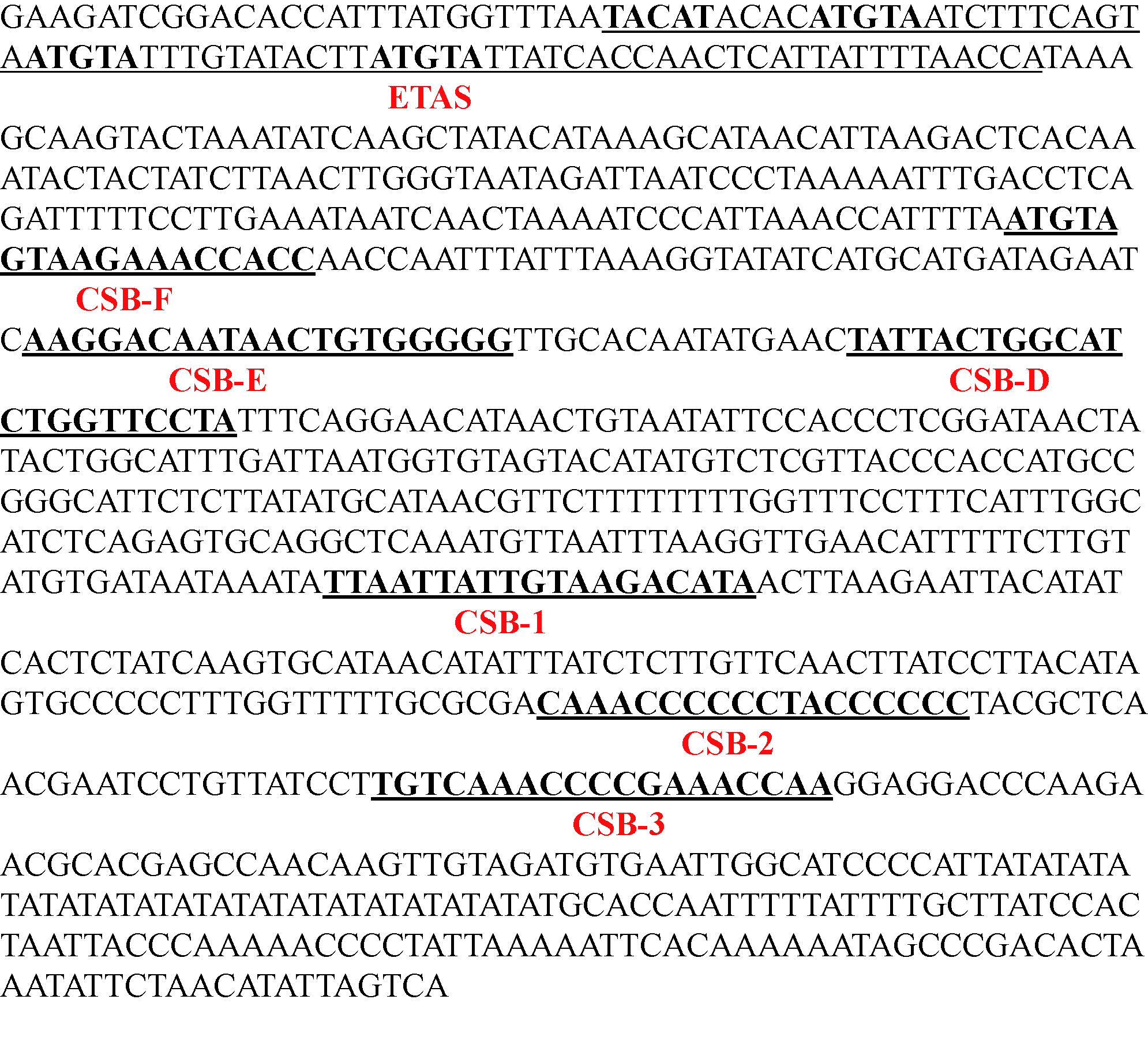

Supplement: Supplementary file 1 [file genes-14-01227-s001.zip › Figure S2.tif]
